# Effects of nitric oxide-related compounds in the acute ketamine animal model of schizophrenia

**DOI:** 10.1186/s12868-015-0149-3

**Published:** 2015-03-07

**Authors:** Ludmyla Kandratavicius, Priscila Alves Balista, Daniele Cristina Wolf, Joao Abrao, Paulo Roberto Evora, Alfredo Jose Rodrigues, Cristiano Chaves, Joao Paulo Maia-de-Oliveira, Joao Pereira Leite, Serdar Murat Dursun, Glen Bryan Baker, Francisco Silveira Guimaraes, Jaime Eduardo Cecilio Hallak

**Affiliations:** Department of Neurosciences and Behavior, Ribeirao Preto School of Medicine, University of Sao Paulo, Av Bandeirantes 3900, CEP 14049-900 Ribeirao Preto, Brazil; Center for Interdisciplinary Research on Applied Neurosciences (NAPNA), USP, Ribeirao Preto, Brazil; Department of Biomechanics, Ribeirao Preto School of Medicine, Medicine and Rehabilitation, USP, Ribeirao Preto, Brazil; Department of Surgery and Anatomy, Ribeirao Preto School of Medicine, USP, Ribeirao Preto, Brazil; Brain Institute, Universidade Federal do Rio Grande do Norte, Natal, Brazil; Department of Psychiatry (NRU), University of Alberta, Edmonton, Alberta Canada; Department of Pharmacology, Ribeirao Preto School of Medicine, USP, Ribeirao Preto, Brazil; National Institute of Science and Technology in Translational Medicine (INCT-TM – CNPq), Ribeirao Preto, Brazil

**Keywords:** Sodium nitroprusside, Glyceryl trinitrate, Methylene blue, Ketamine model, Schizophrenia, Therapeutic treatment, Preventive treatment

## Abstract

**Background:**

Better treatments for schizophrenia are urgently needed. The therapeutic use of the nitric oxide (NO)-donor sodium nitroprusside (SNP) in patients with schizophrenia has shown promising results. The role of NO in schizophrenia is still unclear, and NO modulation is unexplored in ketamine (KET) animal models to date. In the present study, we compared the behavioral effects of pre- and post-treatment with SNP, glyceryl trinitrate (GTN), and methylene blue (MB) in the acute KET animal model of schizophrenia.

The present study was designed to test whether acute SNP, GTN, and MB treatment taken after (therapeutic effect) or before (preventive effect) a single KET injection would influence the behavior of rats in the sucrose preference test, object recognition task and open field.

**Results:**

The results showed that KET induced cognitive deficits and hyperlocomotion. Long- term memory improvement was seen with the therapeutic GTN and SNP treatment, but not with the preventive one. MB pretreatment resulted in long-term memory recovery. GTN pre-, but not post-treatment, tended to increase vertical and horizontal activity in the KET model. Therapeutic and preventive SNP treatment consistently decreased KET-induced hyperlocomotion.

**Conclusion:**

NO donors – especially SNP – are promising new pharmacological candidates in the treatment of schizophrenia. In addition, we showed that the potential impact of NO-related compounds on KET-induced behavioral changes may depend on the temporal window of drug administration.

## Background

Schizophrenia treatment represents a major challenge. We have recently reported in a randomized, placebo-controlled trial, a safe, rapid, and long-lasting improvement of positive, negative, anxiety, and depressive symptoms in patients with schizophrenia after a single intravenous injection of the nitric oxide (NO) donor sodium nitroprusside (SNP) [1]. SNP was also effective in clozapine-refractory schizophrenia [2]. In the rat model of schizophrenia using phencyclidine (PCP) injection, pretreatment with SNP abolished behavioral changes associated with positive symptoms, such as hyperactivity and stereotyped behavior [3]. Although no alterations in prepulse inhibition (PPI) of the acoustic startle response after SNP treatment have been reported in naïve rats [4], a recent study showed that SNP increased PPI response in naïve mice, and SNP pretreatment blocked PPI disruption induced by amphetamine [5].

Despite contradictory results in PPI when analyzed in rats or in mice, SNP produced an unequivocal improvement in schizophrenic patients [1,2] and in animal models [3,5]. The exact antipsychotic mechanism of action of SNP is unclear, but it may act through the glutamate N-methyl-D-aspartate (NMDA) receptor-NO-cyclic guanosine monophosphate (cGMP) pathway, which is altered in schizophrenia [6]. In addition, there is evidence of interaction between SNP/NO and the dopaminergic system [7,8].

Glyceryl trinitrate (GTN) is another NO donor. Unlike SNP, which once in the blood stream dissociates immediately and releases NO [9], GTN is able to react with reduced thiols, cysteine derivatives, ascorbate and mitochondrial aldehyde dehydrogenase, yielding bioactive nitrites and NO [10]. On the other hand, methylene blue (MB), a phenothiazine compound routinely used to inhibit soluble guanylate cyclase (sGC) and isoforms of NO synthase (NOS), inactivates NO by generation of superoxide anions [11]. Interestingly, MB pretreatment shows antipsychotic-like properties in the MK-801 mouse model [12].

Sub-anesthetic doses of ketamine (KET), a non-competitive NMDA receptor antagonist, elicit positive and negative symptoms, and cognitive deficits in humans [13] and animal models [14,15]. Single or repeated KET administration induces changes in systems involving glutamate, dopamine, serotonin and gamma-aminobutyric acid (GABA), neurotransmitters implicated in schizophrenia pathophysiology [16]. Glutamate and dopamine altered neurotransmission are particularly involved in positive and negative symptoms, and in cognitive deficits observed in schizophrenia [17], which makes the acute KET model useful for explaining this impairments [15].

The role of NO in schizophrenia is complex and still unclear. Antipsychotic-like properties have been demonstrated in animal models using either pretreatment with NO donors [3,5] or with NO inhibitors [12,18]. NO modulation is unexplored in KET animal models to date, although hippocampal increase of NADPH-diaphorase stained neurons with no changes in neuronal NOS expression has been reported in the sub-chronic KET rat model [14]. In the present study, we compared the behavioral effects of pre- and post-treatment with SNP, GTN or MB in the acute KET animal model of schizophrenia.

## Methods

### Animals

The procedures followed guidelines from the Brazilian Council for Control of Animal Experimentation, and were approved by our local Commission for Ethics in Animal Experimentation (University of Sao Paulo School, Ribeirao Preto School of Medicine; protocol #102/2012). We used 2 month-old male Wistar rats weighing 250-280 g at the beginning of the experiments. Rats were housed in pairs within standard plastic cages before the beginning of the experiments for 1 week after arriving in our facility, and alone during the period when the sucrose preference test was performed, as detailed bellow. Animals had free access to food and water. The colony room was maintained at 21-24°C and on a 12 h:12 h light-dark cycle (lights on at 7 a.m.).

### Drugs and experimental timeline

**Figure 1:**
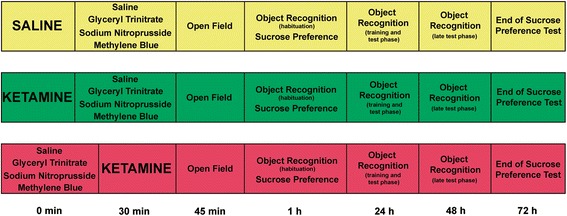
**Timeline of the experimental procedures.** Times depicted represent the beginning of each event with regard to time 0.

Twelve groups of rats (n = 12 to 14 per group, total of 150) were formed according to the injection scheme showed in Figure 1. A maximum of 12 animals (~3 per group) were handled in each 72 hours of experiment in order to assure that all initial injections occurred between 8 and 9 a.m. and that subsequent behavioral tests happened within a very similar time window. Saline (0.9% w/v, Isofarma, Eusebio, Brazil) was administered intraperitoneally (i.p.) in a volume of 0.1 ml/100 g body weight. KET (30 mg/kg, Ketamin S(+), Cristalia, Itapira, Brazil) was administered i.p. in a volume of 1 ml/100 g body weight (dose based on Keilhoff *et al.* [14]). GTN (10 mg/kg, Tridil, Cristalia, Itapira, Brazil) was administered i.p. in a volume of 0.1 ml/100 g body weight (dose based on Pardutz *et al.* [19]). SNP (5 mg/kg, Nitropruss, Cristalia, Itapira, Brazil) was administered i.p. in a volume of 0.1 ml/100 g body weight (dose based on Bujas-Bobanovic *et al.* [3]), protected from light until injection. MB (4 mg/kg, Azul de Metileno P.A., Synth, Diadema, Brazil) was administered i.p. in a volume of 0.4 ml/100 g body weight (dose based on Riha *et al.* [20]). KET was freshly dissolved in saline before each experiment. GTN and SNP were freshly dissolved in 5% (w/v) glucose solution (Isofarma, Eusebio, Brazil) before each experiment. MB was freshly dissolved in milliQ water.

Initially, 4 groups were injected with saline at time 0 and treated with saline, GTN, SNP or MB 30 minutes later (Figure 1, yellow boxes). Four other groups were injected with KET at time 0, and post-treated with saline, GTN, SNP or MB 30 minutes later (Figure 1, green boxes). Four other groups were pretreated with saline, GTN, SNP or MB at time 0 and then injected with KET 30 minutes later (Figure 1, light red boxes). Behavioral tests started 15 minutes after the last injection, as shown in Figure 1. Intervals between injections were defined based on the assumption that we planned to treat the ketamine injected animals during the peak of their positive-like symptoms manifestation, which occurs around 30 minutes after ketamine injection. In consequence, groups on the therapeutic scheme (Figure 1, green boxes) were evaluated (in the open field, for instance) right after this peak, while the groups on the preventive scheme (Figure 1, light red boxes) were still under the acute effect of ketamine. Even though groups on the therapeutic and on the preventive scheme were not in the exactly same time window during open field evaluation, they were both equally under the most prominent effects of ketamine: cognitive deficits and correlates of positive symptoms (please see more details in Results).

### Behavioral tests

#### Sucrose preference

Preference was examined by placing one animal per cage for 72 h with free access to food and with two identical graduated water bottles randomly allocated in the cage (changed after each 24 h), one containing 250 ml of tap water and the other 250 ml of 1% w/v sucrose (Synth, Diadema, Brazil) in tap water. The final volume of each bottle was measured at the end of 24 hours, during 3 consecutive days. Total fluid intake during 72 h in all groups was not different from SAL + SAL (Kruskal-Wallis Dunn’s Q < 2.124, p > 0.06) or among all groups (Kruskal-Wallis Dunn’s Q < 3.160, p > 0.05). Thus, sucrose preference was calculated as the ratio of sucrose intake to total fluid intake and values converted to percentage. Anhedonia, an important component of depressive symptoms, can be assessed in rats by the loss of preference of sweetened water over regular water [21]. In schizophrenia, anhedonia is part of the negative and mood symptoms admixture [22].

#### Object recognition

The object recognition task was performed as described elsewhere [23]. In brief, 20 min habituation with no objects in the arena took place 65 min after the first injection. The next day, training (interaction with two identical objects during 5 minutes) and a test phase 30 min later (one of the objects was replaced by a new object with different shape) occurred 24 hours after the first injection. The following day, 48 hours after the first injection, a “late test phase” was performed, where the animal was allowed to explore one object known since the training phase and a different new one (different from the new object presented in the test phase). A rat was always placed into the center of the arena. Measurements of interaction with each of the objects were taken. Percentage of time spent in exploring the new as compared with the familiar object was used. After each session, the arena and objects were cleaned thoroughly with 20% ethanol to ensure that behavior of the rats was not guided by odor cues. The new object recognition task is sensitive to disruption by NMDA receptor antagonists with good predictive validity, and it is useful to access cognitive dysfunction in schizophrenia models [24].

#### Open field

Locomotor activity was monitored for 20 minutes within an open-field square arena (46 × 46 cm) with a white floor bounded by transparent acrylic walls (Insight, Ribeirao Preto, Brazil). Infrared motion sensors recorded the locomotion and non-ambulatory activities (rearing: upward motion either with forepaws in contact with the wall or freestanding and measured as number of attempts), and the values were averaged in 4 min blocks or shown as total scores over 20 min. Velocity was also measured and yielded similar results (data not shown) as those for distance. Changes in locomotion patterns in the open field, like hyperactivity (increased vertical and horizontal activity) are commonly interpreted as psychotic-like behaviors [25]. Measures of anxiety-like behavior included the relative proportion of trajectory spent exploring the center quadrants relative to those located adjacent to the walls of the arena [26]. The open field arena was divided-by black lines-into 64 squares of 5.75 × 5.75 cm. The central 16 squares were defined as the central zone. Symptoms of anxiety are a central feature of schizophrenia since its characterization [22]. Rats were tested only once on the open field task, and were initially placed into the center of the arena.

### Data analysis

Data were analyzed using the statistical program SigmaPlot (version 11) and SPSS (version 19). Groups were compared using analysis of variance (one-way ANOVA, with Bonferroni *post hoc* test) or Student T test for variables with normal distribution, and Kruskal-Wallis Analysis of Variance on Ranks (with Dunn *post hoc* test) or Mann-Whitney Rank Sum Test for variables without normal distribution. Statistical significance was set at p < 0.05, and values presented as mean ± standard error of mean.

## Results

### Sucrose preference test

**Figure 2:**
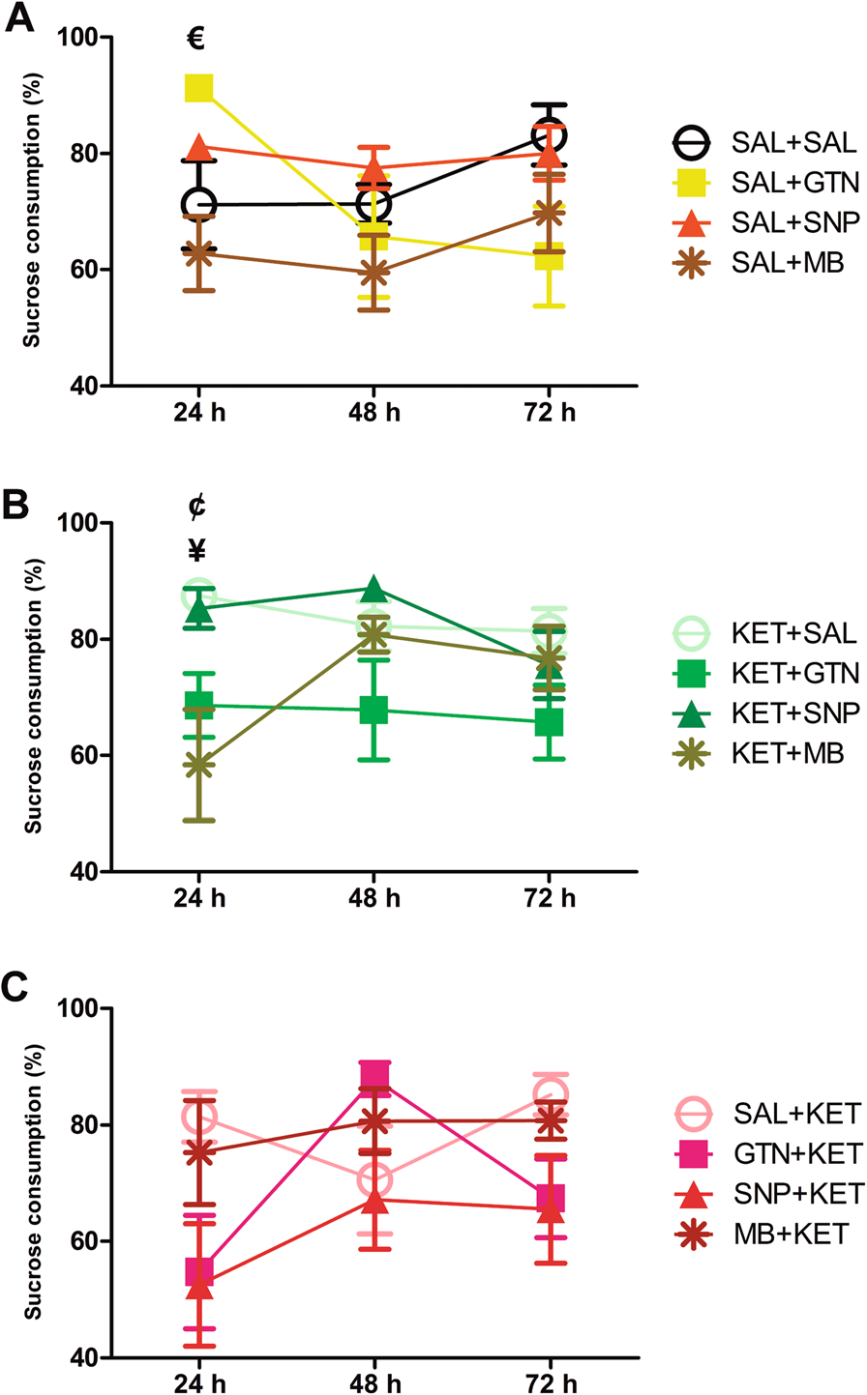
**Percentage of sucrose solution consumption over a 72 hours period.** There was an increase in sucrose consumption in SAL + GTN compared to SAL + MB (€: p < 0.05) in the first 24 h in control animals **(A)**. In the KET model, therapeutic treatment **(B)** with GTN (¥: p < 0.05) and MB (¢: p < 0.05) resulted in anhedonia in the first 24 h. Preventive treatment **(C)** did not result in significant anhedonia. Values presented as mean ± standard error of mean. N = 12 to 14 on each group.

Significant signs of anhedonia were not detected in control animals after GTN, SNP or MB injection, except for an increase in sucrose consumption in SAL + GTN compared to SAL + MB in the first 24 h (Q = 3.275, p < 0.05; Figure 2A). In the KET model, therapeutic treatment with GTN (Q = 2.731, p < 0.05; Figure 2B) and MB (Q = 2.674, p < 0.05) resulted in anhedonia in the first 24 h when compared to KET + SAL. Pretreatment (Figure 2C) with GTN, SNP or MB did not result in anhedonia in the KET model.

### Object recognition task

**Figure 3:**
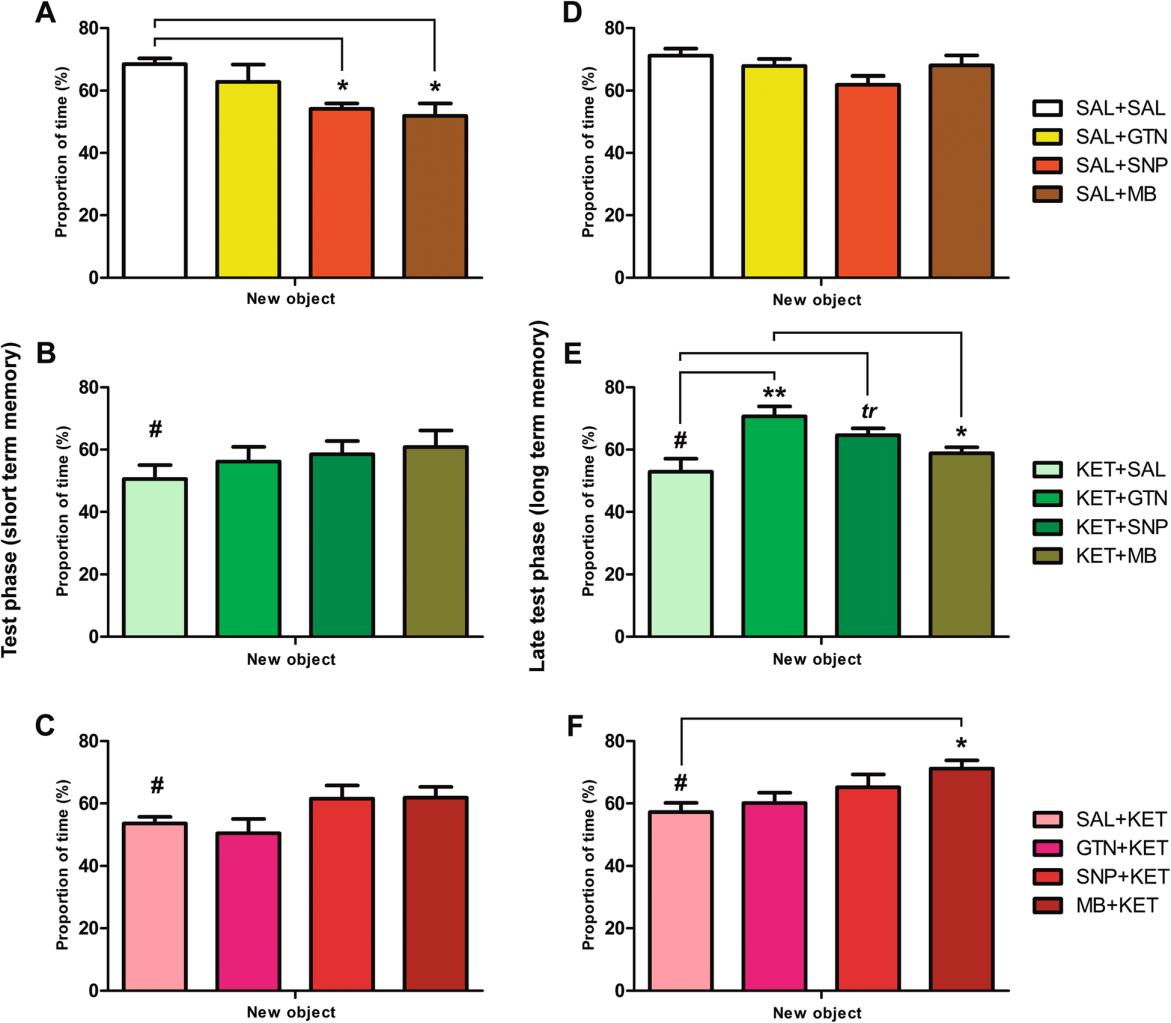
**Object recognition memory.** Test phase (30 min after first object exposition) and late test phase (24 hours after first object exposure) refers respectively to equivalents of short term **(A, B** **and C)** and long term memory **(D, E** **and F)**. SAL+ SNP and SAL + MB showed impaired short term object recognition memory. KET + SAL and SAL + KET animals showed impaired short and long term memory when compared to SAL + SAL (#: p < 0.05). Long term memory improvement was seen in KET animals post-treated with GTN and with SNP, but not on those pretreated. Long term memory improvement was seen in KET animals pretreated with MB, but not on those post-treated. *: p < 0.05; **: p < 0.01; *tr*: p = 0.05. Values presented as mean ± standard error of mean. N = 12 to 14 on each group.

Control animals injected with SNP (Q = 2.857, p < 0.05) and MB (Q = 2.873, p < 0.05) showed impaired short term object recognition memory (Figure 3A). Compared to SAL + SAL, animals submitted to the KET model presented with significant impaired short term (T < 5.259, p < 0.001; hash signs in Figure 3B and C), and impaired long term object recognition memory (T < 3.802, p < 0.001; hash signs in Figure 3E and F). Recovery in long term memory was seen in KET animals post-treated with GTN (F = 3.380, p = 0.01; Figure 3E) and with SNP (a trend, p = 0.05), but not on those pretreated. Long term memory improvement was seen in KET animals pretreated with MB (F = 3.353, p = 0.03; Figure 3F), but not on those post-treated.

### Open field test

**Figure 4:**
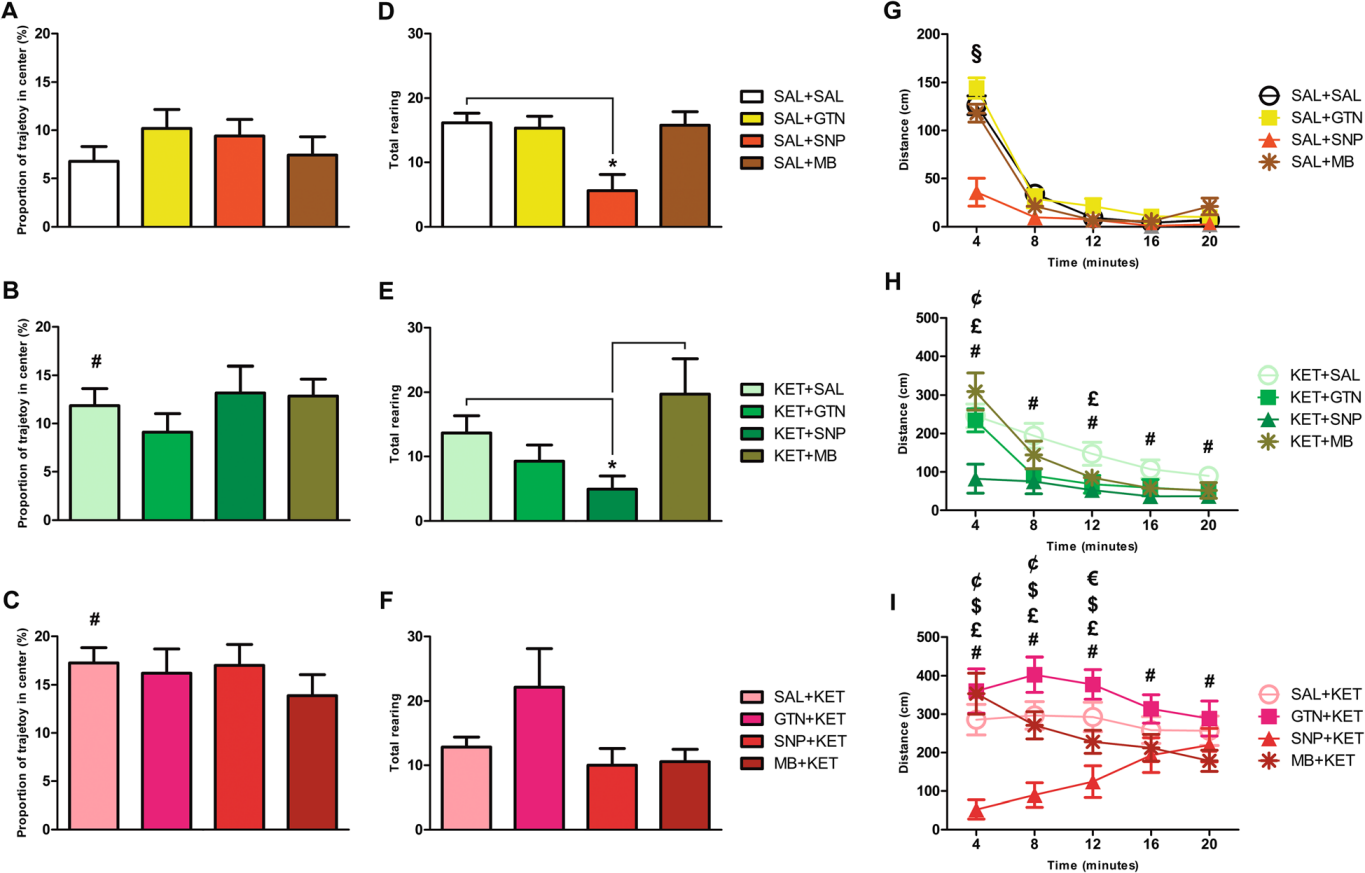
**Behaviors in the open field test.** GTN, SNP or MB did not show anxiolytic effects in control animals regarding the proportion of their trajectory spent in the center **(A)**. KET + SAL **(B)** and SAL + KET **(C)** were more frequently in the center than SAL + SAL (#: p < 0.04). Decreased vertical activity was seen in control animals treated with SNP **(D)** and in KET animals post-treated with SNP **(E)**. Pretreatment did not result in significant differences in vertical activity **(F)**. *: p < 0.05. Horizontal activity was decreased in control animals treated with SNP **(G)** during the first 4 min of the open field test (§: p < 0.05). KET + SAL **(H)** and SAL + KET **(I)** animals presented with hyperlocomotion during all time points of the open field test when compared to SAL + SAL (#: p < 0.003). Therapeutic treatment with SNP was able to revert KET-induced hyperlocomotion (£: p < 0.04 in **H**). KET + MB showed increased horizontal activity when compared to KET + SNP (¢: p = 0.001). Preventive treatment with SNP prevented KET-induced hyperlocomotion (£: p < 0.02 in **I**). GTN + KET ($: p < 0.001) and MB + KET (¢: p < 0.009) showed increased horizontal activity when compared to SNP + KET. GTN + KET showed increased horizontal activity when compared to MB + KET (€: p = 0.03). Values presented as mean ± standard error of mean. N = 12 to 14 on each group.

KET induced an acute anxiolytic effect, probably due to its antidepressant properties, since KET + SAL and SAL + KET animals spent increased proportion of their open field trajectory in the center when compared to SAL + SAL (T < -2.180, p < 0.04; hash signs in Figure 4B and C). No additional anxiolytic effect of GTN, SNP or MB was seen. Control animals injected with SNP showed decreased vertical activity when compared to SAL + SAL (Q = 2.913, p < 0.05; Figure 4D). Therapeutic treatment with SNP of KET-injected animals also resulted in decreased vertical activity (Q = 3.026, p < 0.05; Figure 4E). KET + MB showed increased vertical activity when compared to KET + SNP (Q = 3.533, p < 0.05). Preventive treatment had no effect, although GTN pretreatment resulted in an apparent increase in the total number of rearing, an indication of possible stimulant effect (Figure 4F).

Horizontal ambulation decreased in control animals treated with SNP only during the first 4 min of the open field test (Q > 2.753, p < 0.05; Figure 4G). KET-injected animals presented with hyperlocomotion during all time points of the open field test when compared to SAL + SAL (T < -3.721, p < 0.003; hash signs in Figure 4H and I). Therapeutic treatment with SNP in KET-injected animals reverted KET-induced hyperlocomotion (F > 2.895, p < 0.04; Figure 4H). Similarly, pretreatment with SNP prevented KET-induced hyperlocomotion (F > 7.807, p < 0.03; Figure 4I). Interestingly, while GTN therapeutic treatment of KET-injected animals tended to reduce locomotion, GTN preventive treatment increased hyperlocomotion, although not statistically significant when compared to SAL + KET.

## Discussion

Besides reinforcing the role of NO donors – especially SNP – as promising new pharmacological candidates in the treatment of schizophrenia, the present results also assessed for the first time the potential impact of pre- versus post-treatment with these drugs in the KET model. Subtle differences where seen when comparing therapeutic and preventive SNP treatment, and different outcomes in GTN post-treatment and pretreatment. Therapeutic and preventive treatments lead to similar results in several animal models of neurologic disorders. Some examples include the use of MB, retinoic acid, and cyclophosphamide in animal models of Alzheimer’s disease and Parkinson’s disease, ischemia, and Guillain Barrè syndrome [27-30]. By contrast, other drugs do not exhibit the same effect when used as a therapeutic or preventive treatment. Other examples include the use of interleukin-12 in an animal model of airway inflammation [31] or Rolipram in an experimental autoimmune encephalomyelitis model [32]. Treatment with atypical antipsychotics during the prodromal phase has shown promising results in humans and in animal models of schizophrenia [33,34], but clearly the early intervention paradigm in psychotic disorders must be carefully examined during preclinical phases.

Our results showed significant short and long term memory deficits in the acute KET model consistent with findings in human schizophrenia and in animal models [24]. Therapeutic treatment, but not preventive, with SNP and GTN was able to restore long term memory. MB pretreatment of KET-injected animals was effective in long term memory. Interestingly, SNP and MB induced short term memory deficits in control animals. Other studies on the role of NO donors on spatial working memory have shown no effects of S-Nitroso-N-acetylpenicillamine in control animals [35], or of molsidomine and SNP in a novel object recognition test [36], but the highest concentration of SNP used in this study was 5 times lower than in ours. Although it is likely that a lower SNP dose would not induce short term memory deficits in our control animals, Bujas-Bobanovic et al. have already reported that SNP used only in doses higher than 4 mg/kg is able to abolish completely and in a prolonged fashion PCP-induced ataxia, hyperlocomotion and neuronal activation in several brain regions of the rat [3]. Low doses of SNP are not able to cause significant vascular effects in humans [1], but SNP can improve cerebral dynamics in experimental models [37,38]. Although NO donors may facilitate neurotoxic cascades [39], there is also reports on oxidative damage prevention [40]. Detailed neuropathological alterations in schizophrenia models following SNP remain to be investigated.

The preventive antipsychotic-like action of MB has been documented in a mouse model of schizophrenia using a single injection of MK-801 [12], but in our acute KET model MB was ineffective. MB has been successfully used in the treatment of depressive symptoms in bipolar patients [41]. These findings agree with reports that NO inhibitors may exhibit antidepressant effects in animal models [42]. MB has also been used to reverse spatial memory deficits in rats [43], to improve memory in the amnestic mild cognitive impairment rat model [20], and in our KET model MB pretreatment resulted in long term memory recovery. A possible mechanism, as explored by Callaway et al. [43], would be through increased cytochrome c oxidase activity, enabling an increased metabolic capacity for oxidative energy metabolism in the brain that would improve memory retention. As a therapeutic treatment in the KET model, MB did not cause any memory improvement effect, suggesting that further increased energy metabolism might not be helpful when alterations caused by KET are already on course. In fact, frontal hypermetabolic pattern in positron emission tomography imaging using [18 F]deoxyglucose tracer has been demonstrated in unmedicated patients with schizophrenia [44] and in the KET animal model [45].

Although both GTN and SNP are NO donors, their therapeutic and preventive effects were dissimilar. Long-term memory improvement was seen with therapeutic GTN treatment, but not with the preventive one. GTN pretreatment in the KET model increased vertical and horizontal activity, but that was not the case with GTN post-treatment. Therapeutic and preventive SNP treatment consistently decreased KET-induced hyperlocomotion. In healthy human subjects, GTN treatment does not change cognitive functions [46]. In the KET model, which exhibits cognitive deficits [24], NO-related compounds from the therapeutic treatment with SNP and GTN could improve memory deficit consolidation. Improvement of memory acquisition has also been seen in mice when NO production and neuronal NOS expression were induced, and this effect was abolished by a NO inhibitor [47,48]. In addition, neuronal NOS knockout mice show impaired cognitive performance [49]. If SNP or GTN treatments result in alterations in NOS expression and how that could be related to memory performance remain open questions.

In a previous study, Bujas-Bubanovic et al. [3] reported that SNP pretreatment of SAL-injected Sprague-Dawley rats resulted in a trend to decreased ambulation at the beginning of the open field test when compared to animals injected only with SAL. In our study using Wistar rats, decreased ambulation of SAL-injected animals treated with SNP when compared to animals treated with SAL was also only seen during the first 4 min of the open field test. Different sensitivity depending on strain could explain this tenuous difference. For instance, the effects of NO inhibition on reserpine-induced hypolocomotion seem to depend on the mouse strain used [50].

In the KET model, we noted an anxiolytic effect of KET when compared to control animals. Such effect was also reported when KET was used as a treatment in a model of posttraumatic stress disorder [51], and in anxious depressed patients [52]. Given that anxiety is part of schizophrenia symptoms [22], the acute KET model is not the most suitable model to evaluate its expression. Even with this limitation, we found that NO modulation was unable to affect anxiety-related parameters in control or in KET-injected animals. A similar rationale applies to the sucrose consumption test. While anhedonia may be a component of schizophrenia symptomatology [22], KET action as a fast antidepressant [53] would influence the hedonic state. However, our results suggest that therapeutic treatment with SNP in KET-injected animals has advantages for mood stability when compared to GTN and MB post-treatment. Interestingly, symptoms of anxiety and depression were also improved in patients with schizophrenia treated with SNP [1].

Nevertheless, NO inhibitors are frequently used as a preventive treatment in models of major depression [54,55]. If the effect of NO inhibitors is the same when used in a therapeutic scheme remains to be determined. In the evaluation of cognitive parameters, preventive treatment with the NO donors molsidomine and S-Nitroso-N-acetylpenicillamine was successful in the MK-801 model [35,56]. Meanwhile, pretreatment with either a NO donor or a NO inhibitor was effective in the apomorphine model [36]. In a scenario of massive dopaminergic dysfunction, Gourgiotis et al. show that preventive use of NO donors and NO inhibitors might share similar beneficial outcomes on memory deficits due to their actions on two functionally opposing systems [36]. The exact neurochemical scenario of a glutamatergic dysfunction followed or anticipated by NO augmentation is still unknown, but our behavioral analyses favor therapeutic NO increases. Despite most studies with animal models of psychiatric conditions perform their evaluations using the preventive scheme and have thus contributed with valuable findings, in the clinical practice the therapeutic approach is more frequent. In the acute KET model, pre and post-treatment with GTN, SNP and MB were dissimilar in all behavioral instances analyzed, reassuring the importance of comparing treatment schemes in light of what is transposable to clinical trials.

## Conclusion

In summary, our results using SNP in the acute KET model of schizophrenia reproduced and expanded the findings of the preventive effects described for the PCP model [3]. Also, SNP produced a marked and positive outcome when used as a therapeutic treatment, as also recently highlighted by clinical observations [1,2]. The effects of MB and GTN were more variable, suggesting that the involvement of NO-cGMP/sGC pathway-related molecules in schizophrenia is more complex than initially thought. Additional studies to better understand the differential mechanisms of preventive and therapeutic action of SNP and GTN in animal models may open new venues for treatment of schizophrenia.
